# The elusive life cycle of scyphozoan jellyfish – metagenesis revisited

**DOI:** 10.1038/srep12037

**Published:** 2015-07-08

**Authors:** Janja Ceh, Jorge Gonzalez, Aldo S. Pacheco, José M. Riascos

**Affiliations:** 1 Universidad de Antofagasta, Instituto de Ciencias Naturales Alexander von Humboldt, Climate Change Ecology Group, CENSOR Laboratory, Av. Universidad de Antofagasta 02800, Antofagasta, Chile; 2 Murdoch University Perth, School of Veterinary and Life Sciences, Murdoch, WA 6150, Australia

## Abstract

Massive proliferations of scyphozoan jellyfish considerably affect human industries and irreversibly change food webs. Efforts to understand the role of jellyfish in marine ecosystems are based on a life cycle model described 200 years ago. According to this paradigm the pelagic medusae is considered seasonal and alternates with the benthic polyp stage from which it derives. However, we provide evidence that a) the occurrence of several species of medusae is not restricted to a season in the year, they overwinter, b) polyp- and medusa generations are neither temporally nor spatially separated, and c) “metagenesis” which is defined as the alternation between sexual and asexual generations does not always occur. Hence we recommend additions to the current model and argue that the scyphozoan life cycle should be considered multi-modal, rather than metagenetic. The implications of these findings for jellyfish proliferations, including possible consequences and associated environmental drivers, are discussed.

In recent decades dramatic increases and temporal shifts in jellyfish abundance have been reported from coastal areas around the globe, with equally dramatic effects on ecosystem functions and services^1^,^2^,^3^. The conception that jellyfish blooms are a consequence of anthropogenic activities and the associated sensationalist headlines in the mass media^4^ have prompted jellyfish to become a symbol for the fragility and the declining health status of the world’s oceans. However, not all gelatinous zooplankton bloom and it is primarily species within the class Scyphozoa that play a key role in these problematic proliferations^5^.

The most conspicuous and therefore most recognized appearance of scyphozoans is the pelagic medusa, however, the life cycle of most scyphozoans is characterized by a progression of alternating generations with a temporal and spatial separation of reproductive modes (Fig. 1); it comprises such drastic transformations of the animals’ body plan that scyphozoan polyps, strobilae and ephyrae were originally believed to be different and unrelated animals. Only when Agassiz fully documented the account of the discovery of the life cycle of two scyphozoan species (*Aurelia aurita* and *Cyanea capillata*), a joint work of several naturalists that included the alternation between sexually reproducing pelagic medusae and sessile asexual polyps, and the distinct progression of the animal’s appearance through the life cycle, was this perception corrected^6^. Since Agassiz’s (1860) depiction of the Metagenetic Life Cycle; MLC (Fig. 1), it remains the fundamental model for our understanding of scyphozoan ecology: the MLC is believed to reflect the highly seasonal environment scyphozoans live in^7^,^8^. When resources are abundant and environmental conditions are favourable for growth and reproduction, a motile, pelagic life form (medusa) is maintained whereas when resources are limiting and environmental conditions downgrade, of a sessile, benthic life form (polyp) is adapted^9^. However, interesting exceptions exist: e.g., the holopelagic species *Pelagia noctiluca* develops ephyra directly from planulae and hence lacks a polyp stage^10^, and the genus *Stephanoscyphistoma* skips the medusa stage through direct metamorphosis from ephyra to free swimming planulae^11^,^12^.

In late spring ephyrae are released by strobilae and develop into young medusae, marking the beginning of the pelagic phase. Medusae grow rapidly through summer and reach sexual maturity by fall^5^,^8^,^13^. Scyphozoan medusae are generally considered to live less than one year^8^,^14^. After the sexual reproduction at the end of the pelagic season the medusa population collapses due to a combination of post-spawning senescence, parasite infestation, food limitation, diseases or unfavourable temperatures^5^,^14^,^15^. The settlement of the pelagic planula and the successive metamorphosis into a sessile polyp (scyphistoma) initiates the benthic phase. This phase has previously been described as a resting stage which supposedly functions as a strategy to survive harsh conditions between seasonal intervals of favourable periods^9^. When advantageous environmental conditions return, the polyp is understood to metamorphose from a scyphistoma into a strobila, ready to release ephyrae. This segmentation-like process, called strobilation, proceeds from the apical part of the polyp downwards and results in a stack of disc-shaped segments, called ephyrae, which are released and develop into young medusae. In addition to strobilation scyphistomae are capable of asexually reproducing in various ways and less studied strategies include direct budding of new polyps, the production of stolons and hence new polyps, the production of planuloid swimming buds, longitudinal fission and the formation of chitin-covered cysts; these cysts can survive for long periods of time in less than favourable conditions^16^. The transition between the benthic and the pelagic phase is triggered by changes in temperature, combined with endogenous rhythms of the animal and chemical cues from the environment^13^,^17^,^18^,^19^.

The exact timing of these processes is not well understood but it is likely to be different between species as well as between individuals of the same polyp colony^20^.

The MLC model implies that the occurrence of medusae is restricted to a season in the year and that polyp- and medusa generations alternate and are temporally and spatially separated in scyphozoans.

However, diversions from the MLC model have been indicated in the literature since the model has been published first (Table 1), but these have been treated as odd exceptions to a general pattern and have not been contextually integrated into today’s jellyfish discussion. For example, medusae of various jellyfish species around the globe have been sighted at unexpected times, e.g. in winter^21^. Unusual patterns have also been observed in benthic stages: e.g., with unusual timing for the release of ephyrae^22^. These and other observations suggest that Agassiz’s model does not consistently apply to all scyphozoans and that variations in jellyfish life cycle patterns might be common. Considering that such variations may have huge demographic consequences in these bloom-forming animals, it is critical to review and understand the basic aspects of the scyphozoan natural history.

While conducting a conventional study on population dynamics of the common jellyfish species *Chrysaora plocamia,* examining the seasonal occurrence, body size, sexual maturity and diet composition of medusae in three consecutive years, we found unexpected traits that were difficult to interpret in the light of the MLC paradigm. Therefore, we reviewed a wide range of literature reporting diversions from Agassiz’s model and evaluated the suitability of the model to explain such traits. As a consequence of these findings, we suggest additions to the current MLC model.

## Results

### Seasonal patterns

We observed “rough” seasonal patterns but large differences in the time span of three successive medusa-seasons in Mejillones Bay, (five months, November–March in 2010/11; nine months, November-July in 2011/12 and four months, October-January in 2012/13). Medusae were generally observed from early to late summer (November–February) but the months when medusae occurred in all three years were limited to November, December and January. Apart from these months, animals were recorded as early as October, as late as March and were even observed in April and the winter months June and July. In 2011/12 medusae re-appeared after the summer season in autumn and winter (Figs 2, 3, 4).

### Logistic model

Even though some studies have shown a lack of relationship between the development of gonads and the size of medusae^23^, the relationship between body size and sexual maturity of *C. plocamia* medusae were fitted well to the logistic model (see supplementary Table S1 and supplementary Fig. S5), accordingly, specimen with a bell diameter larger than 40 cm were classified as sexually mature.

### Sexual maturity

High proportions of sexually mature animals were present in all months (Fig. 2, see supplementary Table S2); in fact 50% or more animals were mature in all months, with the exception of March 2010/11 (33%). The first two months of all three medusa-seasons were characterized by the highest fraction of sexual mature individuals (89–100%, Fig. 2), this peak was followed by a decrease throughout the remainder of the season. The lowest proportion of sexually mature animals was usually found at the end of the medusae-season (fraction of sexually mature medusae each year: 33%, 63% and 57%); a chi-square test comparing proportions of mature and immature medusae at the beginning and at the end of each season (the first and last month of the year when medusae were sighted) confirmed that this low proportion was significantly different (2010–2011: *χ*^2^_0.05,1_ = 5.079, *P* = 0.027; 2011–2012: *χ*^2^_0.05,1_ = 18.647, *P* = 3 × 10^−4^ ; 2012–2013: *χ*^2^_0.05,1_ = 4.724, *P* = 0.031).

### Medusa size

According to the ANOVA analyses, the mean medusae size significantly differed between months in each season (2010–11: *F*_*4,563*_ = 127.127, *P* = 3 × 10^−6^; 2011–12: *F*_*5,573*_ = 997.108, *P* = 8 × 10^−9^; 2012–13: *F*_*3,687*_ = 9.601, *P* = 6 × 10^−5^). Post-hoc comparisons indicated that size was always significantly larger at the beginning of the season compared to the end when animals were medium sized (12–39 cm, Fig. 3). Monthly mean sizes ranged from 39.3-16.4 cm in 2010/11, 56.5-2.5 cm in 2011/12 and 41-31.5 cm in 2012/13. Several sizes occurred simultaneously throughout the season, however, small animals (< 12 cm) were only found once in winter (July) 2011/12. When medusae re-appeared in autumn (April) 2011/12, their mean body size, again, was higher than in animals in July before they disappeared, (43.5 and 2.4 cm, respectively) and specimen were sexually mature.

### Medusa diet

The gastric content of *C. plocamia* comprised of pelagic as well as benthic organisms. Even though the proportion of benthic food items varied a lot between different years it was always higher in the beginning of the season (2010/11 8.1-0%; 2011/12 13.4-0%; 2012/13 76.6-36.3%, Fig. 4, see supplementary Table S3) and benthic organisms were not found at all in the last month of two seasons (2010/11 and 2011/12). This decrease was reflected in a Kruskal-Wallis test that showed significant differences between months for the second and third season (*H*_*4*_ = 32.939, *P* = 2 × 10^−5^; *H*_*4*_ = 10.468, *P* = 0.014; respectively), but not for the first (*H*_*4*_ = 6.559, *P* = 0.161, see supplementary Table 3). In 2011/12; when medusae re-appeared in April they contained similar proportions of benthic food as animals in the beginning of the same season (13.4-11.8%). Due to the small size of medusae in July (mean body size 2.4 cm), prey items were indistinguishable thus the food composition was not assessed in this month. One sample was lost in November 2010/11 and one in December 2012/13.

## Discussion

*C. plocamia* medusae in surface waters revealed a number of unexpected life cycle traits that are not captured in the description of the MLC model, or even contradicted the paradigm fully. (1) Animals of various sizes, including sexually mature medusae were present at all sampling times; (2) while large and sexually mature animals prevailed in the population in the beginning of the sampling period, body sizes shifted from large to medium through time and sexual maturity decreased accordingly. (3) The diet composition of medusae included particularly high numbers of benthic organisms in early summer and to a lower degree in all samplings months. Hence, vertical migration to the benthos might be common in *C. plocamia* medusae.

**Figure 1:**
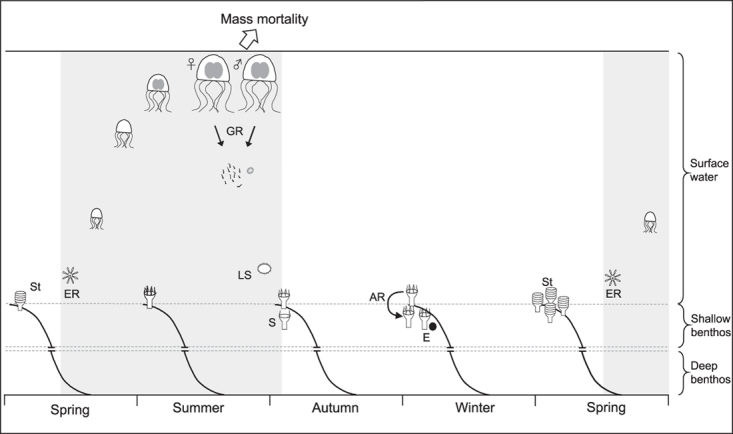
The Metagenetic Life Cycle model, Agassiz (1860). In early spring, ephyrae develop into young medusae. These grow through summer, reach sexual maturity, reproduce sexually and subsequently die. Planula-larvae sink to the seabed, settle and metamorphose into scyphistoma-polyps. Scyphistomae reproduce asexually through strobilation, or produce cysts. In early spring scyphistomae develop into strobilae and release ephyrae. The temporal and spatial separation of the reproductive activity of the polyp- and the medusa generation (benthic and pelagic phase) is represented by white and grey backgrounds, respectively. Sexual maturity is represented by two dark-grey oval structures (gonads). St strobilation, ER ephyrae release, GR gamete release, LS larval stage, S settlement, E encystment, AR asexual reproduction.

**Figure 2:**
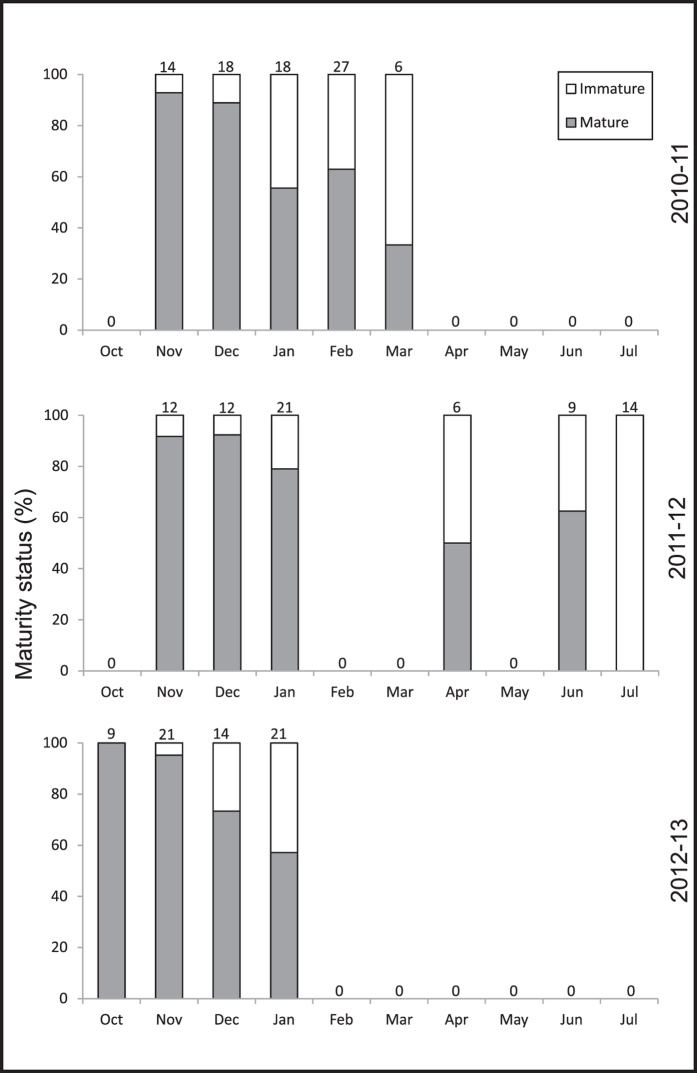
Proportions of mature versus immature *C. plocamia* medusae in three medusa-seasons. Numbers above panels represent the number of animals sampled. Zero values indicate the absence of medusae.

**Figure 3:**
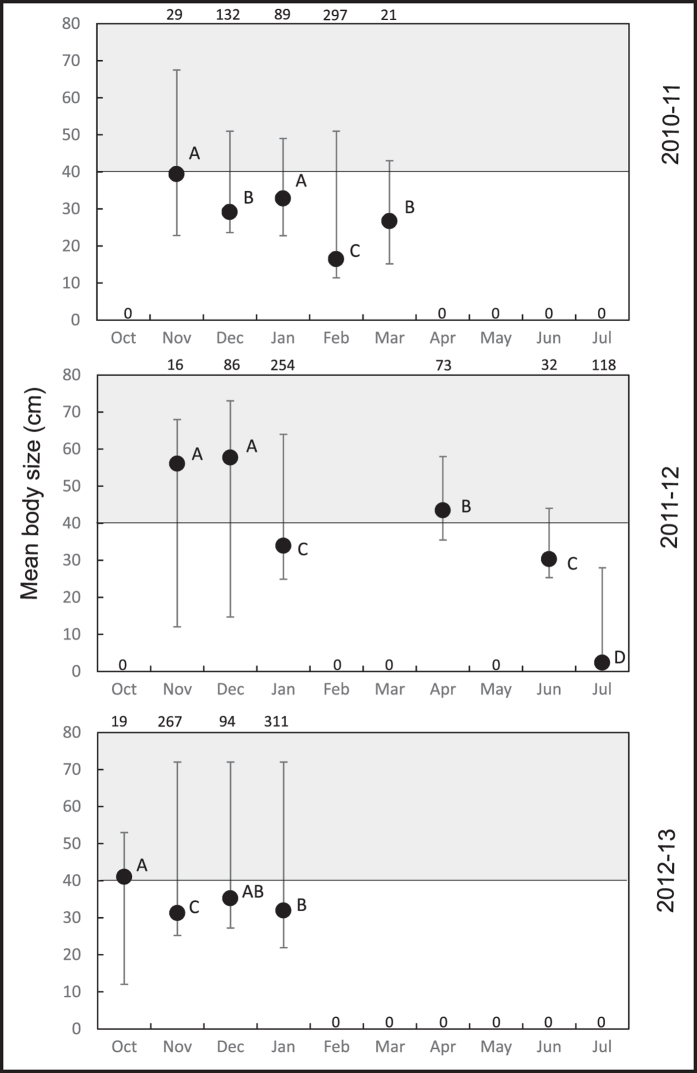
Mean body size of *C. plocamia* medusae in three medusa-seasons. Error bars show the maximum and minimum size. The black line represents the body size above which all animals were expected to be sexually mature (see supplementary Fig. S5). Different capital letters indicate significant differences between months (post hoc comparisons, Tukey-Kramer HSD). Numbers above panels represent the number of animals sampled. Zero values indicate the absence of medusae.

**Figure 4:**
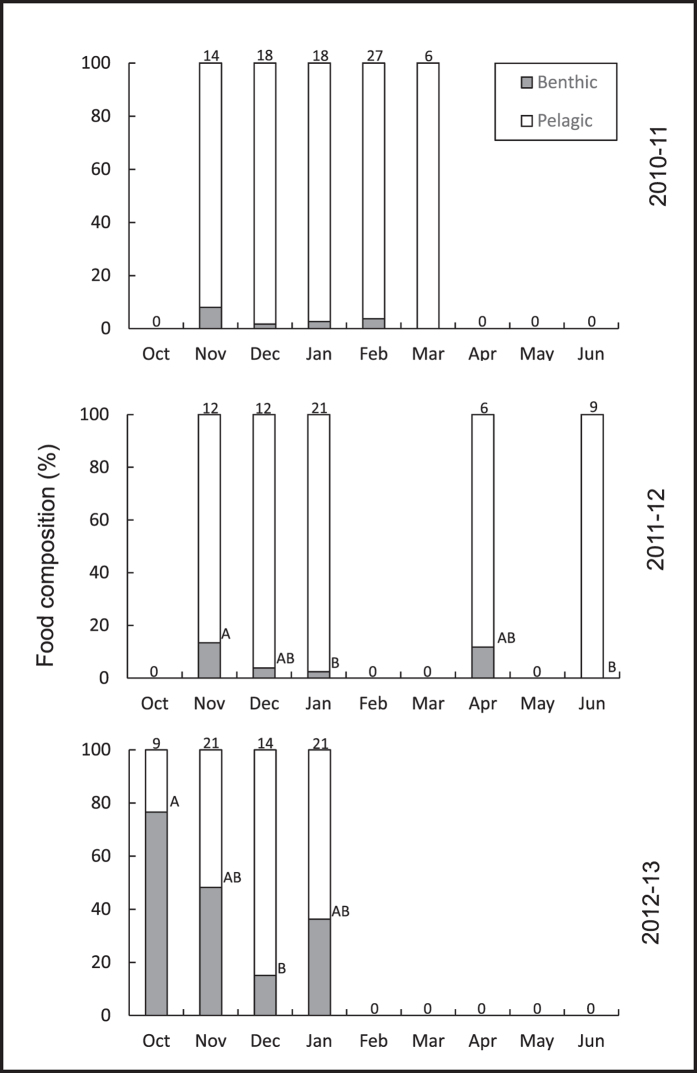
Food composition in gastric pouches of *C. plocamia* medusae in three medusa-seasons. Different capital letters indicate significant differences between months (post-hoc comparisons, Bonferroni correction). Numbers above panels represent the number of animals sampled. Zero values indicate the absence of medusae.

Following the classic MLC model (Fig. 1), the medusa-generation starts with the release of ephyrae from sessile polyps in late spring: medusae appearing in surface waters at this time are expected to be small in size and sexually immature. The following months should be characterized by growth, maturation, reproduction and death at the end of summer. Contrary to the MLC model expectations, *C. plocamia* medusae in surface waters at the beginning of the season were mainly large in size and sexually mature. Both parameters decreased through time and were usually lowest for animals at the end of the season. Individuals of various sizes, sexually mature and immature animals, as well as large dead animals were sighted in surface waters in all months. A protracted release of ephyrae over months is likely and it can be assumed that the medusa-population consisted of multiple cohorts and different ages as opposed to the expected synchronized single cohort described in the MLC model^6^. Interestingly, medusae smaller than 10 cm were never found, other than on a single occasion in winter, suggesting spatial segregation between juvenile and adult medusae, as previously reported^24^.

The medusa-generation of *C. plocamia* displayed a reverse pattern to the one portrayed in Agassiz’s model, i.e., large animals occurred in the beginning and smaller animals at the end of the seasonal occurrence of medusae. These findings raise a plethora of questions forming an interesting basis for new discussions on the suitability of the MLC model as a fundament to study jellyfish biology. For example, where and when do large and sexually mature (*C. plocamia*) medusae grow and mature before they appear in surface waters in late spring? And what happens to small and medium sized medusae that have not reached sexual maturity or reproduced by the end of summer? If, according to the MLC, all medusae die at the end of summer and small animals occur exclusively at the end of spring, how are we to explain the appearance of very small medusae in surface waters in early winter? Interesting clues on the possible whereabouts of large and sexually mature animals prior to appearing in surface waters in late spring were gained from the assessment of medusae-diet. Benthic organisms in gastric pouches of medusae were abundant at all sampling times but highest in late spring: large medusae in the beginning of the season might therefore have emerged from deep waters near the benthos. A consistent presence of benthic food items in the diet might indicate that vertical migrations, that have also been reported for, e.g., the species *P. noctiluca*^10^, are a common behaviour for the medusa-generation of *C. plocamia*. As shown in a recent study, the three most abundant food items in *C. plocamia* medusae, comprising ~88% of their diet, included pelagic eggs of the Peruvian anchovy *Engraulis ringens*^25^, copepods that may migrate from the water column to the sea bed at night time as part of their life cycle^26^,^27^, and the benthic isopod *Excirolana* sp.^28^, (see supplementary Table S4). This diet composition suggests that medusae do not necessarily depend on pelagic resources alone but are able to exploit a wide range of food from different layers in the water column. Benthic assemblages, including the isopod *Excirolana* sp. may be re-suspended by bottom currents, though, or perform active vertical migrations of 1–2 m above the sea floor^29^,^30^, thus becoming an available food source for pelagic animals.

Alternating generations in scyphozoans have been interpreted as an adaptation to food availability and seasonal changes in the environment and winter has been described as an unfavourable time with limited resources when scyphozoans rest in form of benthic polyp stages^9^. In contrast, large *C. plocamia* medusae early in the season contained food items that originated from benthic areas, thus indicating that medusae do survive and feed on benthic or near-bottom prey during “unfavourable” winter times.

**Figure 5:**
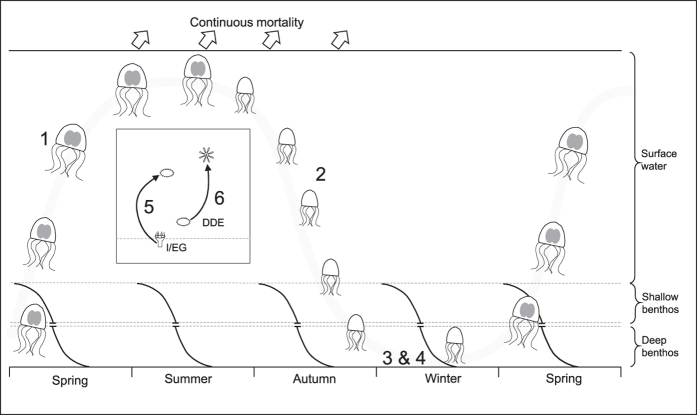
Additions to the present MLC model (Fig. 1), pointing out the multi-modal character of the scyphozoan life cycle. Medusae in surface waters in late spring are large and sexually mature (**1**) whereas animals found later in the season tend to be smaller in size. This observation suggests a constant supply of new medusae due continuous strobilation through summer (not shown). In late summer, medium sized medusae sink to the seabed (**2**), spend the winter near the deep benthos (**3**)/(**4**) and ascent as large and sexually mature animals to surface waters in late spring (**1**). Polyps can produce a mobile planuloid through external/internal gemmation, by-passing the medusa-stage (**5**) and planula-larvae can directly develop into ephyrae (**6**), by-passing the medusa stage. The presented life-cycle-traits have been reported for different scyphozoan species worldwide, not all are necessarily applicable to all species. Grey shadings represent medusae movements. DDE direct development of ephyrae; I/EG internal/external gemmation. Numbers 1–6 follow the numbering in Table 1.

Looking at the clear discrepancies between the MLC model and the medusa-generation of *C. plocamia*, we argue for life cycle traits that are not included in the model as it stands (Fig. 5): our results suggest that the medusa- and polyp-generation are not tied to seasons but can occur simultaneously year round. Small medusae live in close association with the benthos. Not all medusae die at the end of summer, some survive and disappear from surface waters to continue their life in close association with the sea bed; i.e., highly abundant medium size medusae in late summer might rest and grow near the benthos through winter and re-surface as big and sexually mature animals in the following summer season. For *C. plocamia* medusae in Mejillones Bay this notion is supported by antifouling surveillance videos on port structures that frequently recorded medusae in winter but never in summer, usually close to the benthos in approximately 50 meters of depth (E-CL Thermal-Electricity, pers. com. Rodrigo Saavedra). As a consequence *C. plocamia* medusae should be considered pelagic and demersal, rather than pelagic only.

Seasonal variations in the Humboldt Current System are substantially lower than in other environments scyphozoans thrive in and some invertebrates in this system are reproductive throughout the year, albeit with slightly lower and slower reproductive activity during the winter months, e.g., the mollusc *Mesodesma donacium*^31^; this could equally apply to *C. plocamia.* As such, the disclosed mismatch between the MLC model and the results described for *C. plocamia* call into question whether our study describes an exception within the Scyphozoa, possibly related to environmental factors, or an example of a hereto unrecognized general pattern.

To investigate the question, whether and - if so - which diversions from the MLC model have been previously observed, we compiled independent studies from various areas around the globe. This compilation does not include all related literature, many more accounts have been reported, especially for well-studied species like *Aurelia aurita*. We focus our review on scyphozoan life cycle traits missing in the MLC model (Table 1); redundant information was largely excluded.

Section one lists observations of large medusae at the beginning of the medusa-season followed by animals with smaller body sizes through time for *C. fulgida*, *C. plocamia* and *Rhizostoma octopus*^24^,^32^,^33^, similar to findings in the present study. A second set of studies including plankton net as well as surface collection and sonar exploration studies suggests that the life spans of the species *A. aurita*, *Aurelia labiata*, *Catostylus mosaicus* and *C. plocamia* medusae exceed the widely accepted ‘late spring-early autumn period’ by more than double or longer^24^,^34^,^35^,^36^,^37^. Section three consists of trawling studies in deep waters in offshore areas that confirmed the presence of medusae in winter through by-catch, including the species *A. aurita, Chrysaora melanaster, Cyanea capillata* and *R. octopus*^21^,^38^,^39^, sometimes in high numbers^39^.

Most scyphozoan species occur seasonally in coastal surface waters, followed by massive medusae die-offs at the end of the medusa- season^14^. These frequent and impressive events of mortality paired with the simultaneous disappearance of medusae from surface waters have been assumed to mark the end of the medusa-generation. However, observations contradicting the view that all medusae die at the end of the season have consistently been reported and through recurrent observations of medusae in winter the term “overwintering” emerged^33^,^38^,^40^, (Table 1). This term implies that medusae do not die at the end of the season but survive the winter, possibly descending towards deeper water layers. At the same time, however, the term overwintering epitomizes our paucity of knowledge about the fate and behaviour of medusae when they clear out in late summer, as we literally know nothing about their whereabouts and survival strategies in winter. Still, some authors have gone so far as suggesting overwintering as an adaptation to a warming climate^35^,^41^,^42^, that might increase the abundance of medusa-populations^43^. As tempting as such assumptions may be, it is important to note that observations of medusae surviving winter have been reported for about two centuries with accounts from as early as 1837 (in^39^). Accordingly, as the major impacts of climate change have been attributed to the second half of the 20^th^ century^44^, increasing ocean temperatures might facilitate higher numbers of medusae to survive but are unlikely to be the cause of the overwintering phenomenon as such.

Taken together the studies compiled within sections 1–4 in Table 1 reveal that medusae of several scyphozoan species outlive one season, start the season as large animals and occur in deep waters in winter. Thus, they not only support our observations on unknown life cycle traits in *C. plocamia*, but also indicate that the migration of medusae to deep waters in winter is likely to represent a key aspect of the scyphozoan life cycle, rather than an exception. Seasonal migrations in scyphozoans have previously been hypothesized by Boero^10^,^45^ for *P. noctiluca* in the Mediterranean Sea; i.e., jellyfish overcome warmer summer months at colder mid-water levels, migrate upward for sexual reproduction by mid-autumn or early winter and feed on the seasonal spring plankton bloom at shallow levels throughout spring.

Astonishingly, such life cycle traits have previously escaped the attention of authors in a substantial number of studies. One possible explanation for the lack of recognition of these life cycle traits might be that the majority of our knowledge on scyphozoan ecology has been gathered from studies on medusae sampled in coastal surface waters only, whereas most of the studies listed in Table 1 were conducted either in environments of shallow water depths, i.e., from enclosed or semi-enclosed regions, or by methods that allowed the collection of medusae from all depth in deeper waters, i.e., by deep water trawling. These circumstances might have allowed unexpected life cycle traits to be captured, as jellyfish might migrate to deeper waters and thereby ‘escape’ survey efforts conducted in surface waters only.

As a consequence, to better understand jellyfish life cycles, we have to divert away from our conventional ways of studying medusae and expand our research times and tools: i.e., study medusae outside of times when they occur in surface waters and adjust our sampling methods accordingly. To further explore overwintering habitats and survival strategies of medusae we need to track the movement of these animals in winter or even throughout complete years. Submersibles and remotely operated vehicles as well as animal tagging or the use of stable isotope tracers could facilitate such undertakings^46^. Moreover the use of LED blue-light might be useful to find polyps and/or young medusae in their natural environment, taking advantage of their natural fluorescence (where present) as previously suggested for hydrozoans^47^. Boero provides a comprehensive overview of current sampling methods for gelatinous plankton and rightly warns that the absence of focused projects on gelatinous plankton and the inadequacy of the sampling gear to monitor plankton abundance and composition contribute to a widespread lack of reliable information^48^.

Diversions from the MLC model have also been shown for polyps and planulae (Table 1, sections 5 & 6): Vagelli (2007) reported the production of free-swimming propagules via internal and external gemmation by scyphistomae in *A. aurita*. These consecutively develop into new scyphistomae and allow the animal to skip the medusa-generation. The dispersive phase of this species is therefore not restricted to the ephyra-medusa-planula transition, as originally believed^49^. Moreover, *A. aurita* and *Pelagia* sp. can bypass the polyp-generation and produce ephyrae directly from planulae^50^,^51^. Such alternative reproductive strategies may apply to less studied scyphozoan species as well and might affect the propagation of jellyfish immensely.

A solid idea of the scyphozoan life cycle is the prerequisite to understanding the occurrences of jellyfish blooms: life cycle traits reported here make a critical revision of the current MLC model and its associated terms inevitable and we suggest to add on to the model accordingly (Fig. 5). We intend to provide a comprehensive picture of all known life cycle traits and to point out the multi-modal character of the scyphozoan life cycle. As we are likely to discover more life cycle traits in future studies, these additions are to be understood as a work in progress, rather than a paradigm and we strongly encourage contributions and additions for other regions and species. It may be noted that not all reported life cycle traits necessarily apply to all scyphozoan species.

Additions to the current model (Fig. 5):

### Medusae overwinter

Not all medusae die at the end of the medusa-season, they sink to deeper waters, overwinter and re-appear in surface waters as large and sexually mature animals in the following season (Fig. 5).

### Polyps produce ephyrae throughout spring and summer

Differently sized medusae occurring in surface waters throughout the medusa-season indicate the existence of multiple cohorts and therefore the continuous production of ephyrae by polyps, as opposed to the previous belief that polyps produce ephyrae only in spring.

### Polyp- and medusa generations are neither temporally nor spatially separated

Polyp- and medusa-stages can occur simultaneously: for example, polyps produce ephyrae during the “pelagic phase”, while some medusae sink or migrate to deeper waters in late summer and continue their life in deeper waters through the “benthic phase”. As such, the disappearance of medusae in late summer merely marks the end of the medusa-season (the time when medusae occur in coastal surface waters), but not the end of the medusa-generation. Whether medusae overwinter at the same depth as polyps is unknown, however, they temporally co-occur with polyps through all seasons. The terms sexual/asexual and pelagic/benthic in the classical sense of the MLC model therefore become obsolete and medusae surviving winters near the seabed should not be considered solely pelagic but pelagic/demersal.

The term “metagenetic” is defined as the alternation between sexual and asexual generations, however, an alternation between generations does not always occur in scyphozoans, as polyps can generate polyps (asexual-asexual), i.e. through budding and cysts and even by by-passing the medusa generation as a dispersal stage, producing free-swimming planuloids on their own^49^. On the other hand, planulae can produce ephyrae^51^ and thereby produce the next medusa-generation, skipping the asexual polyp-generation (sexual-sexual).

Owing to the dramatic ecological and economic effects of jellyfish blooms there is considerable debate regarding the global long-term trends in jellyfish abundance. While some studies indicate local increases in the frequency and magnitude of population outbreaks to be linked to anthropogenic factors^52^,^53^, more recent evaluations infer that abundance is fluctuating in phase with global interannual environmental oscillations^54^. The evidence underlying this debate consist of 37 datasets on jellyfish abundance obtained from monitory programs that use qualitative scores (e.g., bloom/no bloom), by-catch data, surface collections and nematocyst counts in zooplankton samples^54^. Apart from the limitations of current methods to assess jellyfish abundance^55^, our study shows that current sampling approaches may consistently be missing fundamental traits of the scyphozoan life cycle (e.g., overwintering and vertical migration) that may significantly modify our estimations of jellyfish abundance as medusae in deep waters might not show in such calculations.

Current efforts to understand the role of scyphozoan jellyfish in energy transfer and ecosystem functioning, focus on the medusa generation, based on two assumptions: First, the medusae stock is considered seasonal and is derived annually from the polyp population^56^. Second, somatic growth and turnover rate (production: biomass ratio), the basic parameters to model energy transfer, are usually estimated from size-frequency analyses, a widely used method in fish ecology^57^. However, we provide evidence that medusae of several species live longer than a year; thus their seasonal abundance in surface waters does not necessarily depend on the production of ephyrae but also on the survival success through winter. In addition medusae might be produced year-round, either by polyps or directly from planulae. This implies that medusae should be parameterised in models as year-round consumers, also considering benthic prey as part of their diet. Moreover, trophic interactions and the physiological processes associated to the overwintering of medusae in deep waters are unknown, particularly their ability to shrink under unfavourable conditions or low food availability. Finally our findings show that jellyfish do not display single, seasonal cohorts growing through time as the book-depiction of the life cycle suggests, but multiple cohorts that may not be feasible to follow using length-frequency analysis. Therefore alternative analytical methods are needed to estimate somatic growth and secondary production of these animals.

## Materials and Methods

### Sampling site and study organism

This study was undertaken in Mejillones Bay. This important upwelling area in Northern Chile (23° S) substantially contributes to total fisheries in the country, due to its high levels of biological productivity^58^. Our study organism was the large (up to 1 m bell diameter) and conspicuous jellyfish *C. plocamia*^59^. This species is distributed along the South American Pacific as well as the Atlantic coast and has been reported for its negative effects on human industries (e.g. pelagic fisheries and tourism) in the area^60^. In Mejillones Bay the medusa-season of *C. plocamia* generally occurs from late spring to late summer (October to February) and medusae can be found from the sea surface down to 15 meters depth. They aggregate and can be easily seen from the water surface, due to their vibrant coloration.

### Sample collection

We performed monthly surveys through the medusa-season of *C. plocamia* in 2010/11, 2011/12 and 2012/13 to assess medusa presence, body size (bell diameter), sexual maturity and diet. The study area and the coastlines North (up to Iquique) and South (down to Antofagasta) of Mejillones Bay were monitored for medusae by local fishermen throughout the year to ensure the assessment of the complete medusa-season in each year. Animals sighted in surface waters were reported and surveys were performed on the following day when medusae were reported outside of the usual medusa season (October-February). We performed visual surveys from a boat covering the bay up to the 150 m isobath in a zig-zag pattern in 20 minute intervals and collected all animals visible from the water surface down to a depth of 15 meters. Animals were collected from the boat and in depth >2 m by scuba divers, using 80 cm diameter dip nets (1 mm mesh size), and their bell diameter was measured to the nearest 0.5 cm using a metric tape. Due to a strongly differing abundance of medusa through seasons, sample size calculation would not have been useful prior sampling. At each sampling point three randomly chosen individuals were collected in plastic bags and processed on the boat to assess size, reproductive and dietary patterns: the gastric pouch was excised and opened, the stomach content was rinsed through a 100 μm mesh sieve and preserved in 5% borate-buffered formaldehyde solution in sea water within 30 minutes after collection to avoid digestion. The gonad tissue was separated from other tissues and stored on ice.

### Sample processing

Four biopsies of gonad tissue were analysed using a compound microscope (Leica, DM1000, Wetzlar, Germany). Animals were classified as either immature (no mature gonads) or mature (with either ripe testes or ovaries). Food items in gastric pouches were identified to the lowest taxonomic level possible using a dissecting microscope (Motic SMZ-168-TL, Hong Kong). Items were classified as benthic or pelagic.

### Data analysis

In order to assess changes in sexual maturity of medusae we modelled the relationship between sexual maturity and body size using the percentage of mature medusae in each size range from pooled monthly samplings for three seasons (N = 222). These data were fit into a logistic regression model, a widely used practise to analyse this type of relationship (equation (1)):


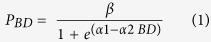


P_BD_ is the fraction of mature male or female medusae in each size range, BD is the bell diameter and *β*, *α1* and *α2* are the parameters. The model was fitted by non-linear least squares, using the Levenberg-Marquardt algorithm to estimate the standard error of parameters. We then plotted the monthly composition of mature animals in each medusa-season to assess the temporal pattern. The model allowed us to estimate the size of sexually mature animals.

Differences between proportions of mature and immature medusae at the beginning and the end of each medusa-season (2010–11: N = 20; 2011–12: N = 26; 2012–13: N = 30) were evaluated using Chi-square 2 × 2 contingency tables, with Haber correction for continuity^61^.

In order to analyse changes in body size in each medusa-season we performed independent 1-way analyses of variance to test for differences in body size between months (2010–11: N = 568; 2011–12: N = 579; 2012–13: N = 591). These models used time (month) as a fixed factor and body size as the dependent variable. Size data were square-root transformed to meet statistical assumptions of normality and variance homogeneity. Post-hoc comparisons were performed by the Tukey-Kramer HSD accounting for differences in sample size and multiple comparisons.

Differences between months in proportion of benthic/pelagic food items were analysed using a Kruskal-Wallis test by ranks (2010–11: N = 83; 2011–12: N = 60; 2012–13: N = 65). As data did not meet the assumptions of an ANOVA model a non-parametric test was used. When significant differences were found, we performed pairwise comparisons of mean ranks for all groups with Bonferroni correction for multiple comparisons. A significance level of α = 0.05 was chosen for all the test performed in this study. All statistical analyses were performed using Statistica 10.0 for Windows (StatSoft, Inc 1984–2011, USA).

## Additional Information

**How to cite this article**: Ceh, J. *et al.* The elusive life cycle of scyphozoan jellyfish – metagenesis revisited. *Sci. Rep.* **5**, 12037; doi: 10.1038/srep12037 (2015).

## Supplementary Material

Supplementary Information

## Figures and Tables

**Table 1:** Scyphozoan life cycle traits missing in the Metagenetic Life Cycle model.

Type of observation & details	Species	Method	Study Area	Source
**1. Larger medusae at the beginning of the pelagic season**				
Large medusae only present in the first sampling month	*Chrysaora fulgida*	Trawling	Benguela, Namibia (O)	32
Body size decreased through the pelagic season	*Chrysaora plocamia*	Surface collections	Northern Chile (C)	This study
Larger animals in summer than in autumn	*Chrysaora plocamia*	Surface collections	Southern Peru (C)	24
Larger animals in early summer than in early autumn	*Rhizostoma octopus*	Strandings	Southern Irish & Celtic Seas (C)	33
**2. Extended life span of medusae**				
Medusae present through a 15-months sampling period	*Aurelia aurita*	Plankton nets	Urazoko Bay, Japan (S)	62
Medusae present through a 1-year sampling period	*Aurelia aurita*	Sonar	Lake Nakaumi, Japan (E)	35
The authors suggest a 2-year life span for medusa	*Aurelia aurita*	Plankton nets	Kagoshima Bay, Japan (S)	36
Medusae/ephyrae present through a 1-year sampling period	*Aurelia aurita*	Plankton nets	Kiel Fjord, Germany (S)	22
Medusae/ephyrae present through a 1-year sampling period	*Aurelia aurita*	Plankton nets	Horsea Lake, England (E)	23
The authors suggest a 2-year life span for medusa	*Aurelia labiata*	Surface collections	Roscoe Bay, Canada (E)	34
The authors suggest a 13-months life span for medusa	*Catostylus mosaicus*	Surface collections	Lake Illawarra, Australia (E)	37
Medusae present through a one year sampling period	*Chrysaora plocamia*	Surface collections	Independencia Bay, Peru (C)	24
**3. Medusae present in deep waters in winter**				
Medusae caught in fishing nets in winter	*Aurelia aurita*	Trawling	United Kingdom (O)	39
Medusae caught in trawls in late winter	Chrysaora melanaster*	Trawling	Bering Sea (O)	21
Large medusae caught in trawls in autumn/winter	*Cyanea capillata*	Trawling	North Sea (O)	38
Large medusae caught in trawls in winter	*Cyanea capillata*	Trawling	Gullmard Fjord, Sweden (S)	63
Specimen has been registered in winter at times	*Rhizostoma octopus*	Trawling	United Kingdom (O)	39
**4. Medusae overwinter**				
Overwintering as a climatic adaptation	*Aurelia aurita*	Sonar	Lake Nakaumi, Japan (E)	35
Overwintering as a climatic adaptation	*Aurelia aurita*	Not reported	Seto Island, Japan (C)	41
Ephyrae overwintered in deep water	*Aurelia aurita*	Trawling	Gullmard Fjord, Sweden (S)	40
Overwintering causes increased abundances of medusa	Chrysaora melanaster*	Trawling	Bering Sea (O)	43
Large medusae overwintered	*Cyanea capillata*	Trawling	North Sea (O)	38
Overwintering inferred from year-round strandings	*Rhizostoma octopus*	Strandings	Southern Irish/Celtic Seas (C)	33
**5. In/externally produced free-swimming propagules**	*Aurelia aurita*	Laboratory	Not reported (L)	49
**6. Development of ephyrae from planulae**				
Observed in rearing experiments	*Aurelia sp.*	Laboratory	Not reported (L)	50
Planulae developed ephyrae and polyps	*Aurelia aurita*	Laboratory	Urazoko Bay, Japan (L)	51
Observed in rearing experiments	*Pelagia sp.*5	Laboratory	Not reported (L)	50

Type of environment: (E), (S), (C) = enclosed, semi-enclosed, coastal water bodies; (O) = offshore waters; (L) = laboratory. * > 80% of biomass corresponds to *C. melanaster*.
