# Supplementary material for: The elusive life cycle of scyphozoan jellyfish – metagenesis revisited
Source: Sci Rep. 2015 Jul 8;5:12037. doi: 10.1038/srep12037 (PMC4495463; doi:10.1038/srep12037)

Manuscript Title: The elusive life cycle of scyphozoan jellyfish – metagenesis revisited

Authors: Janja Ceh, Jorge Gonzalez, Aldo S. Pacheco, José M. Riascos

Supplementary Table S1: Parameter estimates and associated statistics of the logistic model (S5) describing the relation between body size and sexual maturity.

Parameter	Estimate	Standard error	t-value (df=10)	p-value
B	0.098	0.019	50.083	3×10^{-8}
α1	15.855	2.860	5.554	9×10^{-5}
α2	0.551	0.100	5.547	1×10^{-4}

Supplementary Table S2: 1-way analysis of variances: differences in body size of *C. plocamia* between months for each medusa-season (Fig. 2).

	Source	df	Sum of Squares	F-ratio	Prob>F
2010-11	Month	4	18.313	127.127	3×10^{-6}
	Error	563	20.276		
	Total	567	38.590		
2011-12	Month	5	171.083	977.108	8×10^{-9}
	Error	573	20.065		
	Total	578	191.148		
2012-13	Month	3	1.990	9.601	6×10^{-5}
	Error	687	47.475		
	Total	690	49.46		

Supplementary Table S3: Kruskal-Wallis testing differences between benthic/pelagic food items (Fig. 4) in the content of gastric pouches of *C. plocamia*.

	N	Df	H	P
2010-11	83	4	6.559	0.161
2011-12	60	4	32.939	2×10^{-5}
2012-13	65	3	10.468	0.014

Supplementary Table S4: Mean abundance of food items (number per medusa) in gastric pouches of *Chrysaora plocamia* in three medusa-seasons in Mejillones, Northern Chile. Food items were classified according to previous studies [1-3].

Food item	Mean abundance (standard error)			
	2010-2011 N=83	2011-2012 N=60	2012-2013 N=65	Average
**PELAGIC**				
Engraulis ringens eggs	33.36 (8.10)	65.73(46.34)	315.26(114.30)	138.12
Appendicularia	0.00(0.00)	0.00(0.00)	7.14(4.24)	2.38
Fish eggs	0.98(0.22)	13.83(5.55)	2.51(0.57)	5.77
Siphonophora	0.00(0.00)	0.00(0.00)	1.32(0.46)	0.44
Ctenophora	0.00(0.00)	0.00(0.00)	0.25(0.15)	0.08
**PELAGIC, TOP-DOWN MIGRANT**				
Copepoda (Some orders within the copepods can be benthic, active emergent).	43.90(12.17)	75.05(24.19)	0.00(0.00)	39.65
Bivalve larvae	5.65(3.11)	1.49(0.66)	0.49(0.18)	2.55
Polychaete larvae	1.75(1.17)	4.29(2.40)	0.18(0.09)	2.08
Bryozoan larvae	0.31(0.20)	1.98(1.06)	0.00(0.00)	0.76
Cladocera	0.01(0.01)	13.92(8.73)	0.26(0.13)	4.73
Ostracoda	0.01(0.01)	3.44(1.61)	0.12(0.09)	1.19
Ostracoda larvae	0.00(0.00)	0.00(0.00)	0.25(0.11)	0.08
Fish larvae	0.00(0.00)	0.00(0.00)	0.25(0.11)	0.08

Hydromedusae	0.04(0.02)	1.42(1.00)	0.00(0.00)	0.49
Thaliacean salps	0.02(0.02)	0.15(0.15)	0.06(0.06)	0.08
Gastropoda larvae	0.01(0.01)	0.00(0.00)	0.00(0.00)	0.00
Euphausiid	0.01(0.01)	0.00(0.00)	0.00(0.00)	0.00
**BENTHIC**				
Ophiuroidea juveniles	0.00(0.00)	0.22(0.11)	1.66(0.76)	0.63
Malacostraca*: Emerita analoga*	0.01(0.01)	0.00(0.00)	0.00(0.00)	0.00
**BENTHIC, ACTIVE EMERGENT**				
Malacostraca: *Excirolana* sp.	0.09(0.06)	0.83(0.58)	34.62(6.49)	11.84
Cirripedia larvae	0.99(0.49)	0.17(0.11)	0.55(0.21)	0.57
Decapod post-larvae	0.25(0.08)	5.51(2.47)	1.86(0.58)	2.54
Polychaeta	0.06(0.03)	2.97(1.41)	0.06(0.06)	1.03
Amphipoda: *Caprella* sp.	0.04(0.02)	0.00(0,00)	0.00(0.00)	0.01
Isopoda	0.04(0.02)	0.37(0,19)	0.00(0.00)	0.14
Euphausiid: *Nematoscelis* sp.	0.04(0.02)	0.00(0,00)	0.00(0.00)	0.01
Amphipoda	0.00(0.00)	0.19(0,10)	0.00(0.00)	0.06
**BENTHIC, PASSIVE EMERGENT**				
Bivalvia juveniles	0.05(0.03)	0.00(0,00)	0.18(0.18)	0.08
Gastropoda juveniles	0.04(0.02)	0.20(0,15)	0.18(0.10)	0.14
**UNIDENTIFIED ITEMS**	0.20(0.09)	1.24(0,81)	0.25(0.09)	0.56

References:

1 Alldredge, A. & King, J. The distance demersal zooplankton migrate above the benthos: implications for predation. *Mar. Biol.* **84**, 253-260 (1985).

2 Berasategui, A. A., Dutto, M. S., Chazarreta, J. & Hoffmeyer, M. S. Seasonal occurrence and hatching success of benthic eggs of calanoid copepods in the Bahía Blanca Estuary, Argentina. *Marine Biology Research* **9**, 1018-1028 (2013).

3 Pacheco, A. S. *et al.* Moon phase effects and timing of emerging macrobenthic assemblages in a sheltered soft-bottom sublittoral habitat. *Journal of Sea Research* **86**, 34-42 (2014).

Supplementary Figure S5: Logistic regression model showing the relation between sexual maturity and body size in *C. plocamia*. Parameter estimations and associated statistics are given in supplementary Table S1.

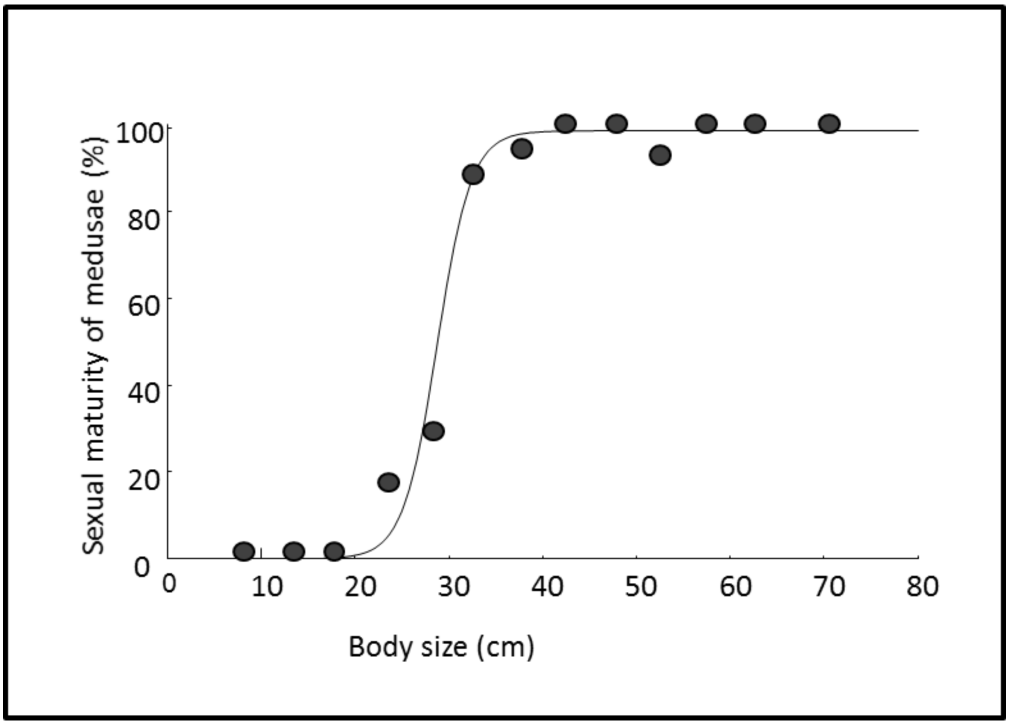

Supplement: Supplementary Information [file srep12037-s1.pdf]
